# Web-Based Harm Reduction Intervention for Chemsex in Men Who Have Sex With Men: Randomized Controlled Trial

**DOI:** 10.2196/42902

**Published:** 2023-01-05

**Authors:** Edmond Pui Hang Choi, Kitty Wai Ying Choi, Chanchan Wu, Pui Hing Chau, Jojo Yan Yan Kwok, William Chi Wai Wong, Eric Pui Fung Chow

**Affiliations:** 1 School of Nursing The University of Hong Kong Hong Kong China (Hong Kong); 2 Sticky Rice Love Hong Kong China (Hong Kong); 3 Department of Family Medicine and Primary Care School of Clinical Medicine The University of Hong Kong Hong Kong China (Hong Kong); 4 Melbourne Sexual Health Centre Alfred Health Melbourne Australia; 5 Central Clinical School Faculty of Medicine, Nursing and Health Sciences Monash University Melbourne Australia; 6 Melbourne School of Population and Global Health The University of Melbourne Melbourne Australia

**Keywords:** chemsex, drug abuse, eHealth, men who have sex with men, trial, prevention, Chinese

## Abstract

**Background:**

Men who have sex with men (MSM) who practice chemsex have a higher likelihood of engaging in risky sexual behaviors and higher rates of HIV infection and other sexually transmitted infections (STIs) than those who do not.

**Objective:**

This trial aimed to evaluate the effectiveness of a web-based intervention in reducing the sexual harms of chemsex among MSM.

**Methods:**

The study was a 2-arm, assessor-blinded, randomized, parallel-group trial with a 3-month follow-up period. The study was conducted in the year 2021 in Hong Kong. Underpinned by the theory of planned behaviors and a harm reduction approach, the intervention consisted of interactive components and knowledge-based information about chemsex. Participants in the control group received brief information and content about sexual violence. The primary outcome was self-efficacy in refusing risky sexual behaviors and chemsex, as measured by the Condom Self-Efficacy Scale (CSES), Self-Efficacy for Sexual Safety (SESS) instrument, and Drug Avoidance Self-Efficacy Scale (DASES). The secondary outcomes included intentions to have chemsex, actual engagement in chemsex, HIV and other STI testing, and condom use in the last 3 months. All outcomes were self-reported. An online structured questionnaire was used to collect data.

**Results:**

In total, 316 MSM enrolled in the study. The intervention group demonstrated a significantly larger improvement in condom-use self-efficacy (as measured by CSES scores; time-by-group interaction: β=4.52, 95% CI 2.03-7.02; *P*<.001), self-efficacy for sexual safety (as measured by SESS scores; time-by-group interaction: β=2.11, 95% CI 0.66-3.56; *P*=.004), and drug avoidance self-efficacy (as measured by DASES scores; time-by-group interaction: β=6.98, 95% CI 1.75-12.22; *P*=.009). Regarding the secondary outcomes, participants in the intervention group demonstrated a significantly larger reduction in the likelihood of having engaged in chemsex in the last 3 months (time-by-group interaction: odds ratio [OR]=0.23, 95% CI 0.10-0.53; *P*=.001) and likelihood of having had the intention to engage in chemsex in the last 3 months (time-by-group interaction: OR=0.37, 95% CI 0.18-0.78; *P*=.009). Participants in the intervention group also showed a significantly larger increase in the likelihood of having undergone HIV testing in the last 3 months (time-by-group interaction: OR=3.08, 95% CI 1.72-5.54; *P*<.001).

**Conclusions:**

This study suggests that a web-based intervention with a harm reduction approach can enhance the self-efficacy of MSM in refusing risky sexual behaviors and chemsex and improve the uptake of HIV testing. We also provide initial evidence that such interventions can reduce both the intention of MSM to engage in chemsex and their actual engagement in chemsex.

**Trial Registration:**

ISRCTN Registry ISRCTN20134522; https://www.isrctn.com/ISRCTN20134522.

**International Registered Report Identifier (IRRID):**

RR2-10.1186/s12889-021-10742-8

## Introduction

“Chemsex” is defined as the use of psychoactive substances before or during planned sex to facilitate, initiate, prolong, sustain, or intensify the sexual encounter [1,2]. There is no universal and systematic definition of types of chemsex drugs because the drugs used might vary based on place and time [3]. Nonetheless, there are 4 substances typically associated with chemsex: methamphetamine, mephedrone, γ-butyrolactone/γ-hydroxybutyric acid, and ketamine [4]. Chemsex occurs across sexual orientations and genders but is considerably more common among men who have sex with men (MSM) [5-7]. A systematic review reported that the prevalence of recent (within the last 6 months) engagement in chemsex among MSM populations is high, with estimates ranging from 9.9% to 93.7% (according to 12 studies) [8]. Motivations for engagement in chemsex vary. A systematic review reported that MSM engage in chemsex because it can increase their stamina and arousal levels, allowing them to engage in sex for sustained periods; the lowering of inhibitions induced by chemsex can also provide a more immediate and sustained interaction with sex partners [2].

Empirical studies have reported that the practice of risky sexual behaviors is prevalent among MSM who engage in chemsex. A systematic review reported that the prevalence of condomless sex ranged from 17% to 100% among MSM who engaged in chemsex [8]. A study in the Netherlands also reported that the participating MSM who engaged in chemsex were significantly more likely to practice condomless anal sex than the MSM who did not (84.3% vs 61.1%, respectively; *P*<.001) [9]. According to a qualitative study conducted in the United Kingdom, MSM perceive condomless sex as the norm during chemsex [10]. Chemsex is also associated with group sex, fisting, and a higher number of sexual partners [8,11,12]. Robust evidence suggests that chemsex is strongly associated with high-risk sexual behaviors linked to the risk of acquisition of HIV and other sexually transmitted infections (STIs).

MSM who engage in chemsex experience higher rates of HIV infection, other STIs, and hepatitis C infection than those who do not [4]. A cohort study in Canada reported that practicing chemsex is linked to an increased incidence of gonorrhea and chlamydia and that this effect is stronger for people who use multiple chemsex substances [13]. A study in the United Kingdom reported that the rate of new HIV diagnoses is significantly higher in MSM who practice chemsex than in those who do not (8.6% vs 1.8%, respectively) [12]. The study also reported that MSM who practice chemsex have higher odds of having a serodiscordant HIV-positive sex partner (adjusted odds ratio [OR]=6.83) [12]. The high frequency of STIs among chemsex users highlights the importance of primary, secondary, and tertiary prevention of STIs and chemsex among MSM.

Globally, “undetectable equals untransmittable (U=U)” and pre-exposure prophylaxis (PrEP) have become important elements in HIV prevention programs [14]. However, the uptake of U=U and PrEP has been limited and slow in the Asia-Pacific region [14]. It was reported that U=U has not yet been widely applied in clinical settings in the Asia-Pacific region to empower people living with HIV to use antiretroviral treatments to achieve and maintain their untransmittable status and to live normal sexual and social lives [14]. However, there has been no systematic study evaluating the use of U=U in Hong Kong. According to the most recent review of the HIV/AIDS situation in Hong Kong, sexual transmission remained the major mode of HIV transmission [15].

In addition, even though PrEP is a very effective HIV prevention method [16], unequal access to PrEP services continues to negatively affect many people who could benefit from it worldwide. It was estimated that just under 1 million people had initiated oral PrEP by 2020, which is far less than the 2020 Joint United Nations Programme on HIV/AIDS target of 3 million [17]. Furthermore, access to PrEP is still highly concentrated in a small number of countries. By the end of 2020, fewer than 20 countries recorded more than 10,000 PrEP initiations [17]. In Asia, which was the source of nearly 20% of the world’s HIV infections in 2019, only a few countries (eg, Thailand and Vietnam) have implemented national guidelines outlining PrEP as an HIV prevention strategy [17,18]. Moreover, another study estimated that Southeast Asia contributes less than 5% of all the PrEP initiations recorded worldwide [19]. Despite increasing scientific evidence on its effectiveness and safety, international recommendations, and the rising global adoption of PrEP in HIV prevention, PrEP is currently not available as a part of public health care services in Hong Kong. People in Hong Kong can only obtain PrEP in the private sector at a very high cost (about HK $8000/month [US $1032]) or purchase it from other countries [20]. In areas where the availability of PrEP is still limited, behavioral interventions to promote safer sexual practices, such as consistent condom use and regular HIV testing, still play an important role in HIV prevention.

eHealth, health services and information delivered electronically through the internet (eg, through a website) [21], has become a commonly used modality for health promotion. A systematic review of the process evaluation of 8 eHealth interventions reported that eHealth interventions targeting sexual risk and substance use are acceptable for MSM across different sociodemographic groups [22]. Another systematic review and meta-analysis of 46 studies supported the effectiveness of eHealth privacy interventions in promoting HIV-preventive behaviors, such as condom use and STI testing, among MSM [23]. Thanks to the well-established internet access that most people have worldwide, one of the most important advantages of eHealth is the high degree of accessibility it offers by granting access to remotely located people [24]. Moreover, compared with face-to-face interventions, eHealth interventions provide greater anonymity, privacy, and accessibility, which are particularly relevant and important for promoting sexual health among MSM.

Although several systematic reviews have provided robust evidence about the effectiveness of eHealth interventions in improving sexual health among MSM [23,25,26], there has been no study related to chemsex and its prevention. To address this knowledge gap, this study aimed to evaluate the effectiveness of a web-based intervention in reducing the sexual harms of chemsex among MSM, with the objectives of strengthening the self-efficacy of MSM in refusing risky sexual behaviors and chemsex, reducing both their intention to engage in chemsex and their actual chemsex behaviors, enhancing consistent condom use during both sober and drug-influenced sex, and increasing motivation toward the practice of regular HIV and STI testing.

We hypothesized that the web-based intervention could increase participants’ self-efficacy in refusing risky sexual behaviors and chemsex, reduce both the intention to engage in chemsex and actual chemsex behaviors, enhance consistent condom use, and increase the uptake of HIV and STI testing.

## Methods

### Study Design

This study was a 2-arm, assessor-blinded, randomized, parallel-group trial with a 3-month follow-up period. The trial protocol has been published [27] and registered. There were no important changes to the methods and study content after trial commencement.

### Study Participants and Setting

The study participants were enrolled using convenience sampling from June 15, 2021, to November 5, 2021. To ensure that we could recruit the required number of study participants from diverse backgrounds, potential participants were approached using social media (eg, Instagram). Local nongovernmental organizations that target MSM populations in Hong Kong also helped recruit potential participants based on their existing networks. Participants were eligible for inclusion in the trial if they self-identified as cis-male MSM aged ≥18 years with internet access and the ability to read and understand Chinese.

### Screening, Baseline Assessment, and Randomization

An online screening questionnaire was used to screen the study participants for eligibility. Eligible participants were asked to sign an electronic consent form and use their email addresses to register for the study. They also needed to set a personal password to gain access to the study intervention. After completing these steps, the participants were asked to complete an online baseline questionnaire. After completing the baseline questionnaire, participants were randomly assigned to either the intervention group or the control group via a computer-generated block randomization procedure (with a block size of 4 with no stratification) with a 1:1 randomization ratio. The computer-generated sequence was created by the independent programmer who developed the online platform. The online platform conducted masking and allocation concealment. All participants and research team members were blinded to the allocation sequence before the allocation. After randomization, the study participants were automatically guided to the web content associated with their allocation.

### Interventions

The intervention was divided into 2 parts. The first part involved an interactive component. Enrolled participants were first invited to complete 2 quizzes to review their level of understanding of chemsex. The quizzes assessed how much the participants knew about chemsex. Each quiz contained 10 multiple-choice questions. The 2 sets of quizzes differed in terms of the level of difficulty. Participants received their score after completing the quiz. In the second part, participants were given knowledge-based information about chemsex and its potential risks and legal consequences. The side effects of different chemsex substances were also covered. Additionally, the participants were presented with information about how they could protect themselves from contracting HIV and other STIs and regarding local resources for emotional support and HIV and STI testing.

The contents of the intervention were developed based on the theory of planned behaviors [28,29]. For example, the interventions aimed to (1) lower participants’ desire to engage in chemsex by enhancing their knowledge of chemsex (eg, regarding its side effects, risks, and legal consequences) and (2) improve the consistency of participants’ condom use by enhancing their attitude toward and knowledge about condom use. Also, the contents of the intervention aimed to intervene from the perspective of harm reduction, primarily with regard to sexual harm. Because it is difficult for current chemsex users to withdraw from drug use over a short period, being empowered to carry out safer sex practices, even in drug-influenced situations, may be a more realistic way to lower the risks associated with HIV and STI transmission. For example, checklists of what to pay attention to before, during, and after chemsex were provided to foster responsible attitudes toward and practices of chemsex. The safety precautions an individual can take to reduce harm related to chemsex practice were also emphasized. Participants in the intervention group were expected to spend only 30 minutes to 45 minutes to complete the overall intervention.

The control group was offered brief information and educational content about sexual violence, which was not relevant to the chemsex intervention component. There was no interactive component in the control group. The details of the intervention and control groups are shown in Multimedia Appendix 1.

Study participants had unlimited access to their allocated content during the study period. However, the content was only available to participants with a registered email address and password to minimize contamination between the intervention and control groups.

### Outcomes

The primary outcome was self-efficacy in refusing risky sexual behaviors and chemsex. As there was no well-established study instrument to specifically assess this primary outcome, the traditional Chinese version of the Condom Self-Efficacy Scale (CSES) [30], Self-Efficacy for Sexual Safety (SESS) instrument [31], and Drug Avoidance Self-Efficacy Scale (DASES) [32] were used to measure the primary outcome.

The CSES is a 14-item instrument covering 3 domains: (1) consistent condom use, (2) correct condom use, and (3) condom use communication. The total score ranges from 14 to 70, with a higher score indicating a higher level of condom use efficacy [30]. The Cronbach alpha coefficient of the CSES was 0.94 in the current sample. The SESS is a 7-item instrument for assessing participants’ confidence in practicing safer sex. The total score ranges from 7 to 35, with a higher score indicating a higher level of self-efficacy for safe sex [31]. The Cronbach alpha coefficient of the SESS was 0.89 in the current sample. The DASES was used to assess drug avoidance self-efficacy. It includes 16 items assessing abstinence self-efficacy across different high-risk situations. The total score ranges from 16 to 112, with a higher score indicating a higher level of self-efficacy for resisting drug use [32]. The Cronbach alpha coefficient of the DASES was 0.89 in the current sample.

The secondary outcomes included (1) an intention to have chemsex in the last 3 months, (2) actual engagement in chemsex in the last 3 months, and (3) HIV and other STI testing in the last 3 months. We assessed and evaluated participants’ intentions to have chemsex in the last 3 months because we wanted to supplement the outcomes relating to their actual engagement in chemsex. It is known that people who intend to have chemsex do not necessarily eventually engage in chemsex. Therefore, their intentions to have chemsex in the past 3 months were assessed. For participants who engaged in chemsex in the last 3 months, data were collected on the (4) practice of condomless sex during non-chemsex in the last 3 months and (5) practice of condomless sex during chemsex in the last 3 months.

All study outcomes were self-assessed at baseline and 3-month follow-up interviews through an online structured questionnaire. The online questionnaire was pilot tested. A trained research assistant would send reminders (via email or instant messaging) to the study participants to ask them to complete the follow-up interview. There was no change to trial outcomes after the study commenced.

### Sample Size

To detect a small-to-moderate between-group difference (Cohen d=0.4) in the primary outcome through an independent samples *t* test and to achieve a power of 80% at a .05 level of 2-sided significance, at least 200 participants (100 participants in each group) were required. Based on a systematic review of eHealth interventions about HIV and STI prevention among MSM [26], we estimated that the dropout rate would be 20%. Therefore, at least 250 participants would need to be recruited. G*Power was used to calculate the sample size.

### Statistical Analysis

Descriptive statistics were used to summarize the sociodemographic characteristics and study outcomes of the participants at each time point. Baseline characteristics and study outcomes (1) between the intervention and control groups and (2) between participants who completed the study and those who did not (dropouts) were compared using Fisher exact tests for categorical variables or an independent samples *t* test for continuous variables.

The intention-to-treat principle was applied. Linear mixed effects models were used to assess the differential change in continuous outcomes (ie, scores on the CSES, SESS, and DASES). Similarly, generalized linear mixed effects models with logit links were used to analyze the binary outcomes (ie, intention to have chemsex, actual engagement in chemsex, undergone HIV testing, undergone other STI testing, practice of condomless sex during non-chemsex, and practice of condomless sex during chemsex in the last 3 months). Time, group, and interaction between group and time were included as independent variables. Mixed effects models can accommodate missing data and do not require imputation of missing observations, providing a natural way to deal with missing values or dropouts. For the main analysis, no covariates were put in the models. To supplement the main analysis, complete case analysis was conducted. Statistical analysis was performed using SPSS version 25.0 (IBM Corp, Armonk, NY). All statistical tests were 2-tailed, with a 5% level of statistical significance. The data set and analyses were checked by 2 researchers.

### Ethics

The study was approved by the Institutional Review Board of the University of Hong Kong/Hospital Authority Hong Kong West Cluster (HKU/HA HKW IRB; reference number: UW 20-650). Electronic informed consent was obtained from each study participant. Study information such as aims, nature of the study, and brief overview of the content of the intervention were provided in the consent form. This trial followed the CONSORT-EHEALTH statement (Multimedia Appendix 2) and the International Conference of Harmonisation Guidelines for Good Clinical Practice. Study data were de-identified. Only the research team had access to the study data. We established a hotline and email account for enquiries and support.

## Results

**Figure 1:**
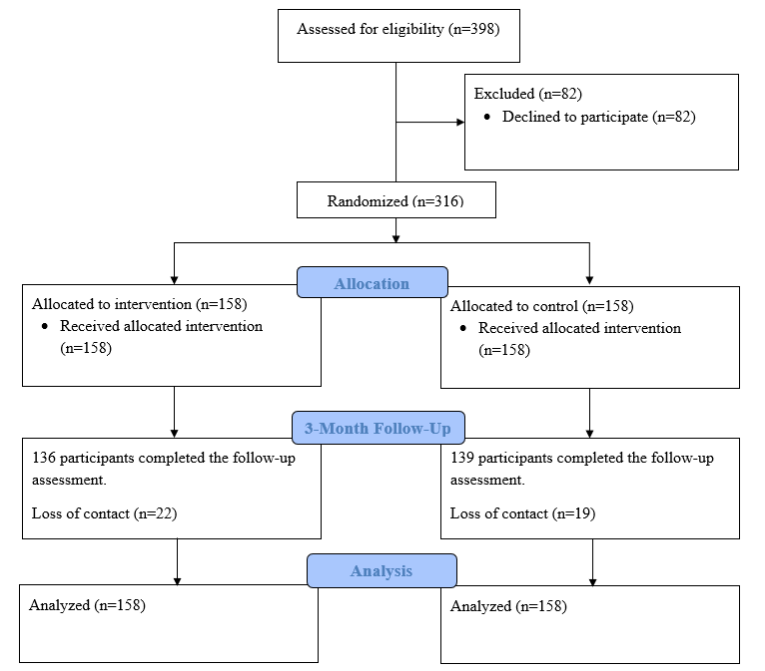
CONSORT flow diagram.

In total, 316 participants were enrolled and randomized into intervention (n=158) and control (n=158) groups. However, 41 participants (22 participants in the intervention group and 19 participants in the control group) did not complete the follow-up survey. The overall dropout rate was 13.0%, (41/316), and there was no significant difference in the dropout rate between the intervention group and control group. Figure 1 shows the CONSORT flow diagram.

### Participant Characteristics

Table 1 shows the study participants’ baseline characteristics. The mean age was 27.34 (SD 6.77) years. Of the 316 participants, 265 (83.9%) were homosexual, and 51 (16.1%) were bisexual. Regarding relationship status, 172 (54.4%) of the 316 participants were in a relationship or married, and 144 (45.6%) participants were single. Of the 316 participants, 199 (63.0%) were employed full-time, and 117 (37.0%) were not employed full-time. Regarding income, 167 (52.8%) of the 316 participants had a monthly personal income less than HK $20,000, and 149 (47.2%) had a monthly personal income of at least HK $20,000.

In total, 104 (32.9%) of the 316 participants had undergone HIV testing in the last 3 months, while 64 (20.3%) of the 316 participants had undergone other STI testing in the last 3 months. Regarding chemsex, 57 (18.0%) of the 316 participants had intended to have chemsex in the last 3 months, while 84 (26.6%) of the 316 participants had engaged in chemsex during their lifetime and 51 (16.1%) of the 316 participants had engaged in chemsex in the last 3 months.

No significant heterogeneity of the demographic data and study outcomes was found between the intervention group and control group, except for relationship status and HIV testing in the last 3 months. No significant heterogeneity of the demographic data and study outcomes was found among those who completed the study (non-dropout) versus those who did not complete the study (dropouts), except for sexual orientation and chemsex in the last 3 months (Multimedia Appendix 3).

**Table 1:** Baseline characteristics and study outcomes of the study participants.

Characteristics and outcomes				Total (n=316)		Intervention group (n=158)		Control group (n=158)		*P* value^a^	
**Baseline sociodemographic characteristics**											
	Age (years), mean (SD)		27.34 (6.77)		27.78 (6.98)		26.90 (6.55)		.25		
	**Sexual orientation, n (%)**										.76
		Homosexual	265 (83.9)		134 (84.8)		131 (82.9)				
		Bisexual	51 (16.1)		24 (15.2)		27 (17.1)				
	**Relationship status, n (%)**										.009
		In a relationship/married	172 (54.4)		74 (46.8)		98 (62.0)				
		Single	144 (45.6)		84 (53.2)		60 (38.0)				
	**Educational level, n (%)**										.77
		Bachelor degree or above	261 (82.6)		129 (81.6)		132 (83.5)				
		Less than a bachelor degree	55 (17.4)		29 (18.4)		26 (16.5)				
	**Employment status, n (%)**										.99
		Employed full-time	199 (63.0)		99 (62.7)		100 (63.3)				
		Not employed full-time	117 (37.0)		59 (37.3)		58 (36.7)				
	**Monthly personal income (HK $), n (%)**										.65
		≥20,000	149 (47.2)		77 (48.7)		72 (45.6)				
		<20,000	167 (52.8)		81 (51.3)		86 (54.4)				
	**Chemsex (lifetime), n (%)**										.90
		Yes	84 (26.6)		41 (25.9)		43 (27.2)				
		No	232 (73.4)		117 (74.1)		115 (72.8)				
**Primary outcomes**											
	Self-Efficacy for Sexual Safety Scale, mean (SD)		25.42 (6.07)		25.13 (6.08)		25.70 (6.07)		.41		
	**Condom Self-Efficacy Scale, mean (SD)**										
		Consistent Use subscale	11.65 (2.82)		11.54 (2.82)		11.75 (2.82)		.51		
		Correct Use subscale	23.87 (4.86)		23.87 (4.74)		23.87 (4.98)		.99		
		Communication subscale	19.15 (4.38)		19.01 (4.45)		19.28 (4.32)		.59		
		Total score	54.66 (11.12)		54.43 (11.04)		54.90 (11.24)		.71		
	Drug Avoidance Self-Efficacy Scale, mean (SD)		83.97 (20.81)		81.91 (21.27)		86.03 (20.20)		.08		
**Secondary outcomes**											
	**Had chemsex in the last 3 months, n (%)**										.76
		Yes	51 (16.1)		27 (17.1)		24 (15.2)				
		No	265 (83.9)		131 (82.9)		134 (84.8)				
	**Intended to have chemsex in the last 3 months, n (%)**										.99
		Yes	57 (18.0)		28 (17.7)		29 (18.4)				
		No	259 (82.0)		130 (82.3)		129 (81.6)				
	**Underwent HIV testing in the last 3 months, n (%)**										.006
		Yes	104 (32.9)		40 (25.3)		64 (40.5)				
		No	212 (67.1)		118 (74.7)		94 (59.5)				
	**Underwent other STI^b^ testing in the last 3 months, n (%)**										.21
		Yes	64 (20.3)		27 (17.1)		37 (23.4)				
		No	252 (79.7)		131 (82.9)		121 (76.6)				
	**Had condomless sex during non-chemsex in the last 3 months (n=51)^c^, n (%)**										.49
		Yes	49 (96.1)		25 (92.6)		24 (100)				
		No	2 (3.9)		2 (7.4)		0 (0)				
	**Had condomless sex during chemsex in the last 3 months (n=51)^c^, n (%)**										.11
		Yes	47 (92.2)		23 (85.2)		24 (100)				
		No	4 (7.8)		4 (14.8)		0 (0)				
**Study dropouts**											
	**Participants who completed the follow-up interview, n (%)**										.74
		Yes	275 (87.0)		136 (86.1)		139 (88.0)				
		No	41 (13.0)		22 (13.9)		19 (12.0)				

^a^Fisher exact test for categorical variables or independent *t* test for continuous variables.

^b^STI: sexually transmitted infection.

^c^Only participants who engaged in chemsex in the last 3 months answered the questions about condom use. At the baseline assessment, 51 participants had chemsex in the last 3 months.

### Primary Outcomes

The effects of the intervention on the study outcomes are summarized in Table 2. Regarding the SESS, the intervention group demonstrated significantly larger improvement in self-efficacy for sexual safety (time-by-group interaction: β=2.11, 95% CI 0.66-3.56; *P*=.004). The intervention group also demonstrated significantly larger improvement in condom use self-efficacy (as measured by their total CSES score; time-by-group interaction: β=4.52, 95% CI 2.03-7.02; *P*<.001) and drug avoidance self-efficacy (as measured by the DASES; time-by-group interaction: β=6.98, 95% CI 1.75-12.22; *P*=.009). The results of the CSES subscale scores are shown in Multimedia Appendix 4.

**Table 2:** Mixed effects models for comparison of study outcomes.

Outcomes				Intervention group							Control group							Group × time effect, β^a^ or OR^b,c^ (95% CI)			*P* value^d,e^
				Results, mean (95% CI)^a^ or n (%)^b^		Within-group change from baseline, β^a^ or OR^b^ (95% CI)		*P* value		Results, mean (95% CI)^a^ or n (%)^b^			Within-group change from baseline, β^a^ or OR^b^ (95% CI)		*P* value						
**Primary outcomes**																					
	**Self-Efficacy for Sexual Safety Scale^f^**																				
		Baseline	25.13 (24.18 to 26.08)		N/A^g^		N/A		25.70 (24.75 to 26.65)			N/A		N/A		N/A			N/A		
		Follow-up	28.59 (27.69 to 29.49)		3.46 (2.43 to 4.48)		<.001		27.05 (26.16 to 27.94)			1.35 (0.33 to 2.37)		.01		2.11 (0.66 to 3.56)			.004		
	**Condom Self-Efficacy Scale: total score^h^**																				
		Baseline	54.43 (52.69 to 56.17)		N/A		N/A		54.90 (53.15 to 56.64)			N/A		N/A		N/A			N/A		
		Follow-up	60.48 (58.87 to 62.09)		6.05 (4.28 to 7.82)		<.001		56.43 (54.83 to 58.03)			1.53 (–0.23 to 3.29)		.09		4.52 (2.03 to 7.02)			<.001		
	**Drug Avoidance Self-Efficacy Scale^i^**																				
		Baseline	81.91 (78.67 to 85.16)		N/A		N/A		86.03 (82.79 to 89.28)			N/A		N/A		N/A			N/A		
		Follow-up	91.06 (87.94 to 94.17)		9.15 (5.43 to 12.86)		<.001		88.19 (85.11 to 91.28)			2.16 (–1.53 to 5.85)		.25		6.98 (1.75 to 12.22)			.009		
**Secondary outcomes**																					
	**Had chemsex in the last 3 months**																				
		Baseline	27 (17.1)		N/A		N/A		24 (15.2)			N/A		N/A		N/A			N/A		
		Follow-up	7 (5.1)		0.26 (0.12 to 0.58)		.001		24 (17.3)			1.17 (0.85 to 1.59)		.34		0.23 (0.10 to 0.53)			.001		
	**Intended to have chemsex in the last 3 months**																				
		Baseline	28 (17.7)		N/A		N/A		29 (18.4)			N/A		N/A		N/A			N/A		
		Follow-up	9 (6.6)		0.33 (0.17 to 0.65)		.001		23 (16.5)			0.88 (0.65 to 1.21)		.43		0.37 (0.18 to 0.78)			.009		
	**Underwent HIV testing in the last 3 months^j^**																				
		Baseline	37 (24.2)		N/A		N/A		57 (38.5)			N/A		N/A		N/A			N/A		
		Follow-up	48 (36.6)		1.81 (1.16 to 2.84)		.01		35 (26.9)			0.59 (0.40 to 0.86)		.006		3.08 (1.72 to 5.54)			<.001		
	**Underwent other STI^k^** **testing in the last 3 months**																				
		Baseline	27 (17.1)		N/A		N/A		37 (23.4)			N/A		N/A		N/A			N/A		
		Follow-up	32 (23.5)		1.49 (0.91 to 2.44)		.11		33 (23.7)			1.02 (0.69 to 1.51)		.93		1.46 (0.78 to 2.76)			.23		
	**Had condomless sex during non-chemsex in the last 3 months (n=31)^l^**																				
		Baseline	4 (57.1)		N/A		N/A		22 (91.7)			N/A		N/A		N/A			N/A		
		Follow-up	5 (71.4)		1.88 (0.57 to 6.13)		.29		24 (100)			175.39 (40.00 to 769.70)		<.001		0.01 (0.002 to 0.07)			<.001		
	**Had condomless sex during chemsex in the last 3 months (n=31)^l^**																				
		Baseline	3 (42.9)		N/A		N/A		19 (79.2)			N/A		N/A		N/A			N/A		
		Follow-up	6 (85.7)		8.00 (0.92 to 69.55)		.06		22 (91.7)			2.90 (0.87 to 9.65)		.08		2.76 (0.23 to 32.83)			.41		

^a^Primary outcomes.

^b^Secondary outcomes, with “no” as the reference category.

^c^OR: odds ratio.

^d^Linear mixed effects models for primary outcomes, with the control group as the reference category. Baseline characteristics were not adjusted in the models.

^e^Generalized linear mixed-effects models for secondary outcomes, with logit links that were used to analyze the binary outcomes and the control group as the reference category. Baseline characteristics were not adjusted in the models.

^f^The total score ranges from 7 to 35, with a higher score indicating a higher level of self-efficacy for safe sex.

^g^N/A: not applicable.

^h^The total score ranges from 14 to 70, with a higher score indicating a higher level of condom use efficacy.

^i^The total score ranges from 16 to 112, with a higher score indicating a higher level of self-efficacy to resist drug use.

^j^Participants who reported being HIV-positive at the baseline assessment were excluded from the analysis.

^k^STI: sexually transmitted infection.

^l^Only participants who engaged in chemsex in the last 3 months answered the questions about condom use. At the follow-up assessment, only 31 participants had chemsex in the last 3 months.

### Secondary Outcomes

Participants in the intervention group demonstrated significantly larger reductions in the likelihood to have engaged in chemsex in the last 3 months (time-by-group interaction: OR=0.23, 95% CI 0.10-0.53; *P*=.001) and intention to engage in chemsex in the last 3 months (time-by-group interaction: OR=0.37, 95% CI 0.18-0.78; *P*=.009). Participants in the intervention group also showed a significantly larger increase in the likelihood to have undergone HIV testing in the last 3 months (time-by-group interaction: OR=3.08, 95% CI 1.72-5.54; *P*<.001). However, no significant group-by-time interaction effect was observed on the outcome of other STI testing in the last 3 months (*P*=.23). During the follow-up interviews, only 31 participants reported engaging in chemsex in the last 3 months. A statistically significant group-by-time interaction effect was observed for the outcome of condomless sex during non-chemsex in the last 3 months (*P*<.001). No significant group-by-time interaction effect was observed on the outcome of condomless sex during chemsex in the last 3 months (*P*=.41). The results of the between-group differences in all study outcomes at each time point are shown in Multimedia Appendix 5. Given that there was a statistically significant difference in relationship status between the intervention group and control group at the baseline assessment, an adjusted analysis was conducted (Multimedia Appendix 6). The results of the complete case analysis (n=275) are shown in Multimedia Appendix 7. The results are consistent with those of the main analysis.

## Discussion

### Principal Findings

In this randomized controlled trial, we found that a web-based intervention with a harm reduction approach could enhance the self-efficacy of MSM in refusing risky sexual behaviors and chemsex. The intervention enhanced HIV testing but had no effects on other STI testing. Importantly, we found that the intention of MSM to engage in chemsex and their actual engagement in chemsex can also be reduced by the intervention. However, it should be noted that, given the small sample size of participants who reported engaging in chemsex in the last 3 months during the follow-up interviews, the findings related to condom use might not be reliable and should be interpreted with caution. Nonetheless, the clinical significance and academic merits of the findings are substantial because this study has provided one of the very first pieces of evidence on the effectiveness of eHealth interventions in reducing the sexual harm of chemsex among MSM, which echoes the recent review by Strong and colleagues [33] that called for more action and identified a need for more harm reduction interventions related to chemsex. In addition, one important feature of the trial was that the contents of the intervention were relatively brief. Participants in the intervention group were expected to spend only 30 minutes to 45 minutes to complete the overall intervention. Such digitally delivered brief interventions can be easily disseminated to hard-to-reach populations such as MSM who practice chemsex, in a cost-effective manner.

The intervention in the current trial was developed through a harm reduction approach because most qualitative evidence suggests that many MSM prefer reducing the harm associated with drug use (eg, by learning how to better manage their use) instead of abstaining altogether [33,34]. Furthermore, while highlighting what kinds of tailored interventions can assist in the reduction of harm, Herrijgers and colleagues [35] showed in their schematic overview that MSM were exposed to different degrees of harm before, during, and after chemsex events. To meet the actual needs of MSM, the intervention contents of the current trial were developed in a comprehensive manner, addressing the different aspects of harm related to chemsex [33,35]. We also added interactive content (quizzes and instant feedback) to the intervention because a meta-analysis of 12 studies reported that a new media intervention using interactive components yielded significant effects on condom use, while interventions using only static content did not yield positive effects on enhancing condom use [36].

Compared with the control group, the intervention group demonstrated statistically significant effects on the primary outcomes with a small-to-moderate effect size (in Multimedia Appendix 7) and on most secondary outcomes (ie, HIV testing, intention to have chemsex, and actual engagement in chemsex). Our findings are in line with those reported by a systematic review and meta-analysis of 46 studies, which supported the effectiveness of eHealth interventions in promoting HIV-preventive behaviors among MSM with a small effect size [23]. Coupled with previous studies [23], our study further supports the benefits of eHealth interventions to reduce risky sexual behaviors among MSM, which ultimately decreases the burdens of HIV and other STIs in the MSM population.

Even though PrEP has provided a new means of harm reduction (ie, primary HIV prevention) in the context of chemsex [33], the uptake of PrEP has remained low in some regions, such as Southeast Asia. A study reported that only 1% had ever used PrEP among MSM in Hong Kong [20]. In geographic areas where the availability of PrEP and access to it are still limited, eHealth interventions to promote safer sexual practice and regular HIV testing still play an important role in HIV prevention. Therefore, this trial still has significant public health implications for helping to control the burgeoning HIV epidemic among MSM.

Another important benefit of the current study is that the intervention was found to reduce the participants’ intention to have chemsex and their actual engagement in chemsex. Given that chemsex tends to be hidden and private and that those who practice chemsex are difficult to reach [37], eHealth interventions can provide MSM with a private, anonymous, and easily accessible platform offering supportive care and useful information. The current study provides empirical evidence for governments, health authorities, and other stakeholders to put more resources into delivering chemsex-preventive interventions and care using online platforms. Also, more implementation research is needed to plan how to position and scale up eHealth interventions regarding chemsex alongside other community-based HIV-preventive interventions.

This study found no significant effect on other STI testing, implying that future interventions should strengthen the components intended to encourage MSM to undergo regular STI testing. Nonetheless, the negative result could be explained by the substantial reduction in the availability of sexual health services during the COVID-19 pandemic [38]. A study in Australia reported that chlamydia and gonorrhea testing dropped significantly during the COVID-19 pandemic [39]. Unlike HIV self-testing, which is available through the government, community-based organizations, and registered pharmaceutical stores [40], MSM in Hong Kong might have experienced difficulty undergoing other forms of STI testing during the pandemic. Also, the attitudes and perceptions toward the consequences of other STIs among MSM could have led to the insignificant finding [41]. Specifically, according to a qualitative study among MSM in Hong Kong, the MSM community is less cautious about other STIs because they are curable and treatable [42]. However, given the rising number of other STIs in MSM, there is an urgent need to strengthen STI screening and care for MSM [43].

### Limitations

There are several limitations and implications for further studies. First, social media, such as Instagram, was used to recruit participants. An online screening survey and electronic consent forms were also used in this study. Participants who were not technology-savvy may thus have been excluded in the recruitment phase. Therefore, the study findings may not be applicable to those who are not technology-savvy and those with low eHealth literacy. Second, study participants were not blinded to group allocation. The study might be subject to performance bias, as participant knowledge of group allocation may have affected their behaviors. Third, we only evaluated participants’ intention to have chemsex in the last 3 months. Further studies should assess their intention to have chemsex in the future such as in the next 3 months. Fourth, the study outcomes were measured 3 months after the baseline, so the long-term sustainability of the intervention remains unknown. Trials with a longer follow-up period are needed to evaluate the sustained effect of eHealth interventions in enhancing the self-efficacy of MSM in refusing risky sexual behaviors and chemsex. Also, an analysis of the cost-effectiveness of eHealth interventions for chemsex should be considered. Fifth, we did not record information about the study participants’ engagement, such as the duration or frequency of their visits. These parameters should be recorded in the future to further investigate how study engagement impacts the effectiveness of interventions. Sixth, during the follow-up interviews, the number of people who had engaged in chemsex in the last 3 months was small (n=31), which might have affected the statistical power and precision of some of the secondary outcomes. Seventh, qualitative interviews should be conducted to obtain qualitative feedback from study participants in the future. Finally, similar trials should be developed and tested in other geographic areas to provide more empirical evidence to support the effectiveness of such interventions in enhancing the self-efficacy of MSM in refusing risky sexual behaviors and chemsex.

### Conclusion

This study suggests that a web-based intervention with a harm reduction approach can enhance the self-efficacy of MSM in refusing risky sexual behaviors and chemsex and improve HIV testing in the 3-month follow-up interview. We also provide initial evidence that such interventions can reduce both the intention of MSM to engage in chemsex and their actual engagement in chemsex.

## Appendix

Multimedia Appendix 1 Content of the intervention and control groups.

Multimedia Appendix 2 CONSORT-EHEALTH checklist (V 1.6.1).

Multimedia Appendix 3 Comparisons of baseline characteristics and study outcomes between non-dropout and dropout participants.

Multimedia Appendix 4 Mixed effects models for comparison of subscale scores of the Condom Self-Efficacy Scale.

Multimedia Appendix 5 Mixed effects models for comparison of study outcomes with the results of between-group difference at each time point.

Multimedia Appendix 6 Mixed effects models for comparison of study outcomes with an adjustment of baseline characteristics.

Multimedia Appendix 7 Complete case analysis for comparison of study outcomes (n=275).

## Abbreviations

CSES: Condom Self-Efficacy Scale
DASES: Drug Avoidance Self-Efficacy Scale
MSM: men who have sex with men
NHMRC: National Health and Medical Research Council
PrEP: pre-exposure prophylaxis
SESS: Self-Efficacy for Sexual Safety
STI: sexually transmitted infection
U=U: undetectable equals untransmittable

## Data Availability

The content of the web-based intervention, data sets generated during or analyzed during the current study, and related syntax are available from the corresponding author on reasonable request.
