# Supplementary material for: Web-Based Harm Reduction Intervention for Chemsex in Men Who Have Sex With Men: Randomized Controlled Trial
Source: JMIR Public Health Surveill. 2023 Jan 5;9:e42902. doi: 10.2196/42902 (PMC9893729; doi:10.2196/42902)
Supplement: Multimedia Appendix 1 [file publichealth_v9i1e42902_app1.pdf]

Multimedia appendix 1: Contents of intervention and control group

Intervention group

A training journey was designed for the intervention group to learn about chemsex. The participants were named ‘Ci Fu’, which is the pinyin of the word ‘masters’. This also aligns with the initials of ‘ChemFun’ (CF), the more common term used to describe chemsex in Hong Kong.

The interface, content flow and progress of the intervention were similar to those of the training implemented by martial art masters. Adopting a harm-reduction approach, the enrolled participants were first invited to play two games to review their level of understanding of chemsex. They then had to read seven information pages covering the details of chemsex, how to reduce sexual risks and other health risks related to chemsex practices and the practice of safer sex.

Games

The two games were mini-quizzes on how much the participants knew about chemsex. Each contained 10 multiple-choice questions. The two sets differed in terms of their level of difficulty. When the participants enrolled in the study, they were invited to answer the first set according to their perceived level of understanding of chemsex. They then received a title (*Beginner/Intermediate/Advanced*) according to the number of correct answers they got in the quiz and were recommended to proceed with the information pages. When they finished the other intervention components, they would revisit the game section. They could then repeat the quiz to test how much they had improved or they could attempt the next level.

Information Pages

The information pages contain a wide range of information. They begin with what chemsex is, why people participate in it and its potential risks and legal consequences. The participants were also presented with information related to how they could protect themselves from contracting sexually transmitted infections (STIs) and about local resources for emotional support and STI testing. To enhance the participants’ knowledge of chemsex, lower their desire to use drugs during sex and encourage them to practice safer sex and regular STI testing, the participants were guided to read the following information in the following order:

- ***About Chem Fun***
  On this page, basic information about chemsex was offered. The participants were introduced to the definition of chemsex, why it appeals to some people and how it is perceived in social situations—for example, on dating apps. After presenting the common types of drugs used and how this has changed over the past 20 years, the potential negative impacts on health and relationships, social stigmas and legal consequences were identified.

- **All You Need to Know about Drugs**
  This page contains an introduction to the most common types of drugs people use during chemsex in Hong Kong, such as ice (methamphetamine), γ-Butyrolactone (GBL)/γ-hydroxybutyric acid (GHB), and ecstasy. We provided basic information about each specific drug, how it works in the body, how it can be consumed, the

immediate effect, the side effects/negative impacts on health, the safe dosage, the possibility of developing dependency and interactions with other drugs. A reminder that the substances are considered dangerous under the laws of Hong Kong and a list of the related penalties were also given.

- **Every Second Counts**
  In this section, we present checklists of what to pay attention to before, during and after chemsex to foster responsible attitudes towards the practice and emphasise the possible safety precautions an individual could take to reduce harm. Specifically, a guide to handling an emergency in which someone overdoses and becomes unconscious was included. The basic steps to ensure one's safety and how to follow up with access to emergency medical support or an ambulance were illustrated in detail.

- **When You Need Help**
  A list of supporting organisations in Hong Kong and the services each of them offers was presented on this page. The services range from individual case follow-ups for chemsex users to sexual health information, HIV and other STI testing, crisis support for sexual violence incidents and community support for men who have sex with men. Relevant social media and hotlines for contact were included to allow easier access to help when needed.

- **The Way to Be Safe**
  To enhance condom use self-efficacy in both sober and drug-influenced sex, detailed information about condoms was introduced on this page. The differences between different types of condoms, such as materials and sizes, how to select the suitable size and the proper way to wear a condom, were presented textually and graphically.
  The material focuses on improving the participants' perceived self-efficacy in making informed decisions on different HIV/STI prevention strategies in both sober and drug-influenced sex.

- **STIs 101**
  The ways in which STIs can be transmitted were illustrated to emphasise the importance of condom usage during sex as the only effective precaution for preventing the contact between high-risk body fluids (for example, pre-ejaculatory fluid, semen and blood) and the genitals or other body parts. The most common STIs and their possible symptoms were presented. The list includes HIV/AIDS, syphilis, gonorrhoea, chlamydia, herpes, genital warts, HPV and pubic lice.

- **Get Home Safe and Get Tested**
  To motivate the practice of regular HIV/STI testing, the participants were encouraged to undergo testing whenever they are sexually active and regardless of whether it was a chemsex encounter. The incubation period and window period of each type of STI were highlighted to remind readers of the appropriate time to get tested after unprotected sexual intercourse. At the same time, the differences between the common types of tests available and where to access these services were listed.

The content was designed based on medical information and reviews of interventions around the world. Some examples are the project Touch ChemSex Support Service developed by the Boys' and Girls' Clubs Association of Hong Kong, the Friday/Monday website produced by Terrence Higgins Trust in the UK and the Know the Score website run by the Scottish Government. Prior to enrolment, the intervention was circulated among front-line workers who provide testing services and community support to MSM in Hong Kong for feedback on content accuracy and relevancy to the local community. Next, it was pilot tested with a group of MSM volunteers to ensure that the use of terms and design were appealing to local MSM.

The theory of planned behaviours was used to guide the intervention development, based on the study conducted in Hong Kong by Wang (2020).

Personal attitudes and subjective norms

Irrespective of the participants' previous engagement in chemsex, they were introduced to how others might think about the practice of chemsex from different angles. Participants were given opportunities to develop and reflect on their perceptions of others' attitudes towards chemsex. Both positive and negative attitudes towards chemsex were highlighted in the intervention, such as (i) chemsex can increase sexual pleasure, (ii) chemsex allows people to escape from reality, (iii) chemsex can harm cognitive functions, and (iv) chemsex can increase the risk of having condomless sex. Participants were encouraged to establish their attitudes towards chemsex after becoming more informed of the facts regarding the benefits and risks of the practice.

On the one hand, the intervention described why chemsex was appealing to some people: for example, its effect in terms of enhancing sensation during sexual experience and the formation of a sense of closeness among users. On the other hand, social stigmas related to the practice were presented in the intervention. For example, some people consider chemsex to be equivalent to the traditional use of illicit or psychoactive drugs and treat people who practice chemsex as drug abusers. The intervention also illustrated why practising chemsex was more prevalent among MSM, how MSM communities perceived chemsex, how the general public perceived chemsex, etc.

Perceived behavioural control

The entire intervention aimed at intervening from the perspective of harm reduction. Hence, different components were incorporated so that participants believed that they could have control over their behaviours and that they were capable of reducing potential harm during sex and chemsex.

In addition to identifying what overdosing during chemsex might look like, advice on reducing harm in risky situations and accessing follow-up emergency medical support were illustrated in detail. In addition, a list of local resources available for the MSM community was provided in the intervention. These included post-incident follow-up services for chemsex users, sexual health information, STI testing, crisis support for sexual violence incidents, and general community support.

Furthermore, as one of the core intentions of the programme was to increase the motivation for condom use during both sober and drug-influenced sex, information relevant to the use of condoms and STI prevention was provided. Detailed instructions on the proper selection and use of condoms were given, and participants were also presented with information on how

STIs can be transmitted through unprotected sex and on the possible symptoms of the most commonly contracted STIs. In order to motivate participants to undergo regular STI testing, the intervention highlighted the differences between the common types of tests available and listed where the tests were accessible in the local community.

Reference

Wang, Z., Yang, X., Mo, P. K., Fang, Y., Ip, T. K. M., & Lau, J. T. (2020). Influence of social media on sexualized drug use and chemsex among Chinese men who have sex with men: Observational prospective cohort study. *Journal of Medical Internet Research*, *22*(7), e17894.

Control Group

The control group was offered brief information and educational content about sexual violence which was not relevant to the intervention component on chemsex.

Information Pages

The participants were asked to go through three information pages providing a brief introduction to what sexual violence is, the emergence of image-based sexual violence and tips to protect oneself online.

- **Sexual Violence 101**
  On this page, the myth that sexual violence does not exist among men was debunked. Despite highlighting that sexual violence could happen to different genders, a more comprehensive definition that any sexual act for which consent was not freely given is considered sexual violence was provided. Some sexually abusive actions that commonly happen among MSM were given as an example. We highlighted the perceived difficulties for men and MSM in seeking help after being sexually assaulted. Details about the various local organisations that provide immediate crisis support to sexual violence incidents—and specifically to those who serve male victims—were provided.

- **Image-Based Sexual Violence**
  Behaviours that were considered image-based sexual violence were identified on this page. Specifically, how these behaviours might occur among MSM was described. Some examples are having explicit images taken or distributed nonconsensually, having one’s picture sexually photoshopped and supplemented with false descriptions (such as the person in the picture having contracted HIV/STIs) and receiving unsolicited nude pictures on dating apps. Related laws and general tips on how to prevent being secretly filmed during a sexual encounter and how to communicate with partners about the privacy of intimate images to avoid sextortion after a break-up were mentioned.

- **How to Protect Myself**
  Information published online without one’s consent can be misused by the perpetrator in incidents of sexual violence. Therefore, on this page, we included reminders about how to protect one’s privacy during any online activities and being cautious of suspicious requests for intimate and explicit images.
