# Supplementary material for: Web-Based Harm Reduction Intervention for Chemsex in Men Who Have Sex With Men: Randomized Controlled Trial
Source: JMIR Public Health Surveill. 2023 Jan 5;9:e42902. doi: 10.2196/42902 (PMC9893729; doi:10.2196/42902)
Supplement: Multimedia Appendix 3 [file publichealth_v9i1e42902_app3.pdf]

Multimedia appendix 3: Comparisons of baseline characteristics and study outcomes between non-dropout and dropout participants

		Total (n=316)	Non-dropout (n=275)	Dropout (n=41)	*P* value [a]
**Baseline socio-demographic characteristics**					
Age (years), mean (SD)		27.34 (6.77)	27.45 (6.64)	26.61 (7.65)	0.461
Sexual orientation, n (%)					
	Homosexual	265 (83.86%)	236 (85.82%)	29 (70.73%)	0.022
	Bisexual	51 (16.14%)	39 (14.18%)	12 (29.27%)	
Relationship status, n (%)					
	In a relationship/ married	172 (54.43%)	148 (53.82%)	24 (58.54%)	0.617
	Single	144 (45.57%)	127 (46.18%)	17 (41.46%)	
Educational level, n (%)					
	Bachelor degree or above	261 (82.59%)	227 (82.55%)	34 (82.93%)	1.000
	Less than a bachelor degree	55 (17.41%)	48 (17.45%)	7 (17.07%)	
Employment status, n (%)					
	Employed full-time	199 (62.97%)	176 (64%)	23 (56.1%)	0.386
	Not employed full-time	117 (37.03%)	99 (36%)	18 (43.9%)	
Monthly personal income (HK $), n (%)					
	≥20,000	149 (47.15%)	127 (46.18%)	22 (53.66%)	0.405
	<20,000	167 (52.85%)	148 (53.82%)	19 (46.34%)	
Chemsex (lifetime), n (%)					
	Yes	84 (26.58%)	74 (26.91%)	10 (24.39%)	0.851
	No	232 (73.42%)	201 (73.09%)	31 (75.61%)	
**Primary outcomes**					
The Self-Efficacy for Sexual Safety Scale, mean (SD)		25.42 (6.07)	25.36 (6.16)	25.80 (5.52)	0.662
The Condom Self-Efficacy Scale					
	Consistent Use Subscale, mean (SD)	11.65 (2.82)	11.57 (2.83)	12.17 (2.69)	0.204
	Correct Use Subscale, mean (SD)	23.87 (4.86)	23.88 (4.92)	23.78 (4.42)	0.899
	Communication Subscale, mean (SD)	19.15 (4.38)	19.14 (4.43)	19.20 (4.12)	0.938
	Total score, mean (SD)	54.66 (11.12)	54.59 (11.25)	55.15 (10.34)	0.767
The Drug Avoidance Self-Efficacy Scale, mean (SD)		83.97 (20.81)	84.07 (21.05)	83.32 (19.31)	0.829
**Secondary outcomes**					
Had chemsex in the last 3 months, n (%)					
	Yes	51 (16.14%)	49 (17.82%)	2 (4.88%)	0.039
	No	265 (83.86%)	226 (82.18%)	39 (95.12%)	
Intended to have chemsex in the last 3 months, n (%)					
	Yes	57 (18.04%)	51 (18.55%)	6 (14.63%)	0.666
	No	259 (81.96%)	224 (81.45%)	35 (85.37%)	
Underwent HIV testing in the last 3 months, n (%)					
	Yes	104 (32.91%)	91 (33.09%)	13 (31.71%)	1.000
	No	212 (67.09%)	184 (66.91%)	28 (68.29%)	
Underwent other STI testing in the last 3 months, n (%)					
	Yes	64 (20.25%)	57 (20.73%)	7 (17.07%)	0.681
	No	252 (79.75%)	218 (79.27%)	34 (82.93%)	
Had condomless sex during non-chemsex in the last 3 months (n=51) [b], n (%)					
	Yes	49 (96.08%)	47 (95.92%)	2 (100.00%)	1.000
	No	2 (3.92%)	2 (4.08%)	0 (0.00%)	
Had condomless sex during chemsex in the last 3 months (n=51) [b], n (%)					
	Yes	47 (92.16%)	46 (93.88%)	1 (50.00%)	0.152
	No	4 (7.84%)	3 (6.12%)	1 (50.00%)	
**Study dropouts**					
Participants who completed the follow-up interview, n (%)					
	Yes	275 (87.03%)			
	No	41 (12.97%)			

Note:
[a] Fisher exact test for categorical variables or independent *t* test for continuous variables.
[b] Only participants who engaged in chemsex in the last 3 months answered the questions about condom use. At the baseline assessment, 51 participants had chemsex in the last 3 months.
Abbreviations:
HIV: human immunodeficiency virus; SD: standard deviation; STI: sexually transmitted infection
