# Supplementary material for: Web-Based Harm Reduction Intervention for Chemsex in Men Who Have Sex With Men: Randomized Controlled Trial
Source: JMIR Public Health Surveill. 2023 Jan 5;9:e42902. doi: 10.2196/42902 (PMC9893729; doi:10.2196/42902)
Supplement: Multimedia Appendix 4 [file publichealth_v9i1e42902_app4.pdf]

Multimedia appendix 4: Mixed-effects models for comparison of subscale scores of the Condom Self-Efficacy Scale

	Intervention Group			Control Group						
Primary outcomes										
	**Mean (95% CI)**	**Within-Group Change From Baseline (95% CI)**	***P* value**	**Mean (95% CI)**	**Within-Group Change From Baseline (95% CI)**	***P* value**	**Between-Group Difference at each time point Mean (95% CI)**	***P* value [a]**	**Group × Time Effect β (95% CI)**	***P* value [b]**
**The Condom Self-Efficacy Scale Consistent Use Subscale [c]**										
Baseline	11.54 (11.10 to 11.99)			11.75 (11.31 to 12.19)			-0.21 (-0.83 to 0.42)	0.511		
Follow-up	12.85 (12.44 to 13.25)	1.30 (0.85 to 1.75)	<0.001	11.97 (11.57 to 12.38)	0.22 (-0.22 to 0.67)	0.329	0.87 (0.30 to 1.44)	0.003	1.08 (0.45 to 1.71)	0.001
**The Condom Self-Efficacy Scale Correct Use Subscale [d]**										
Baseline	23.87 (23.11 to 24.63)			23.87 (23.11 to 24.63)			0.01 (-1.07 to 1.08)	0.991		
Follow-up	26.26 (25.54 to 26.98)	2.39 (1.60 to 3.17)	<0.001	24.61 (23.89 to 25.32)	0.74 (-0.04 to 1.52)	0.063	1.65 (0.64 to 2.66)	0.001	1.65 (0.54 to 2.75)	0.004
**The Condom Self-Efficacy Scale Communication Subscale [e]**										
Baseline	19.01 (18.33 to 19.70)			19.28 (18.59 to 19.97)			-0.27 (-1.24 to 0.71)	0.591		
Follow-up	21.38 (20.75 to 22.01)	2.37 (1.65 to 3.08)	<0.001	19.84 (19.22 to 20.47)	0.57 (-0.15 to 1.28)	0.119	1.54 (0.64 to 2.43)	0.001	1.80 (0.79 to 2.81)	0.001

[a] Bonferroni adjustment was used.

[b] *P* values were obtained by linear mixed-effects models. The control group was the reference category in the models.

[c] The subscale score ranges from 3 to 15 with a higher score indicating a higher level of condom use efficacy.

[d] The subscale score ranges from 6 to 30 with a higher score indicating a higher level of condom use efficacy.

[e] The subscale score ranges from 5 to 25 with a higher score indicating a higher level of condom use efficacy.

Abbreviation:

CI: confidence interval
