# Supplementary material for: Web-Based Harm Reduction Intervention for Chemsex in Men Who Have Sex With Men: Randomized Controlled Trial
Source: JMIR Public Health Surveill. 2023 Jan 5;9:e42902. doi: 10.2196/42902 (PMC9893729; doi:10.2196/42902)
Supplement: Multimedia Appendix 5 [file publichealth_v9i1e42902_app5.pdf]

Multimedia appendix 5: Mixed-effects models for comparison of study outcomes with the results of between-group difference at each time point

	Intervention Group			Control Group			Between-Group Difference at each time point Mean (95% CI)	P value [a]	Group × Time Effect β (95% CI)	*P* value [b]
Primary outcomes										
	Mean (95% CI)	Within-Group Change From Baseline (95% CI)	*P* value	Mean (95% CI)	Within-Group Change From Baseline (95% CI)	*P* value				
**The Self-Efficacy for Sexual Safety Scale** [c]										
Baseline	25.13 (24.18 to 26.08)			25.70 (24.75 to 26.65)			-0.57 (-1.91 to 0.77)	0.405		
Follow-up	28.59 (27.69 to 29.49)	3.46 (2.43 to 4.48)	<0.001	27.05 (26.16 to 27.94)	1.35 (0.33 to 2.37)	0.010	1.54 (0.28 to 2.80)	0.017	2.11 (0.66 to 3.56)	0.004
**The Condom Self-Efficacy Scale: total score** [d]										
Baseline	54.43 (52.69 to 56.17)			54.90 (53.15 to 56.64)			-0.47 (-2.93 to 2.00)	0.709		
Follow-up	60.48 (58.87 to 62.09)	6.05 (4.28 to 7.82)	<0.001	56.43 (54.83 to 58.03)	1.53 (-0.23 to 3.29)	0.088	4.05 (1.79 to 6.32)	0.001	4.52 (2.03 to 7.02)	<0.001
**The Drug Avoidance Self-Efficacy Scale** [e]										
Baseline	81.91 (78.67 to 85.16)			86.03 (82.79 to 89.28)			-4.12 (-8.71 to 0.47)	0.078		
Follow-up	91.06 (87.94 to 94.17)	9.15 (5.43 to 12.86)	<0.001	88.19 (85.11 to 91.28)	2.16 (-1.53 to 5.85)	0.250	2.86 (-1.52 to 7.25)	0.200	6.98 (1.75 to 12.22)	0.009
Secondary outcomes										
		Within-Group Change From Baseline odds ratio (95% CI) [f]	*P* value		Within-Group Change From Baseline odds ratio (95% CI) [f]	*P* value	Between-Group Difference at each time point odds ratio (95% CI)	P value [g]	Group × Time Effect odds ratio (95% CI) [f]	*P* value [h]
**Had chemsex in the last 3 months**										
Baseline	27 (17.09%)			24 (15.19%)			1.15 (0.63 to 2.10)	0.647		
Follow-up	7 (5.15%)	0.26 (0.12 to 0.58)	0.001	24 (17.27%)	1.17 (0.85 to 1.59)	0.335	0.26 (0.11 to 0.63)	0.003	0.23 (0.10 to 0.53)	0.001
**Intended to have chemsex in the last 3 months**										
Baseline	28 (17.72%)			29 (18.35%)			0.96 (0.54 to 1.70)	0.884		
Follow-up	9 (6.62%)	0.33 (0.17 to 0.65)	0.001	23 (16.55%)	0.88 (0.65 to 1.21)	0.430	0.36 (0.16 to 0.80)	0.013	0.37 (0.18 to 0.78)	0.009
**Underwent HIV testing in the last 3 months** [i]										
Baseline	37 (24.18%)			57 (38.51%)			0.51 (0.31 to 0.84)	0.008		
Follow-up	48 (36.64%)	1.81 (1.16 to 2.84)	0.010	35 (26.92%)	0.59 (0.40 to 0.86)	0.006	1.57 (0.93 to 2.66)	0.093	3.08 (1.72 to 5.54)	<0.001
**Underwent other STI testing in the last 3 months**										
Baseline	27 (17.09%)			37 (23.42%)			0.67 (0.39 to 1.17)	0.967		
Follow-up	32 (23.53%)	1.49 (0.91 to 2.44)	0.111	33 (23.74%)	1.02 (0.69 to 1.51)	0.929	0.99 (0.57 to 1.73)	0.164	1.46 (0.78 to 2.76)	0.234
**Had condomless sex during non-chemsex in the last 3 months (n=31)** [j]										
Baseline	4 (57.14%)			22 (91.67%)			0.12 (0.01 to 1.02)	0.052		
Follow-up	5 (71.43%)	1.88 (0.57 to 6.13)	0.292	24 (100.00%)	175.39 (40.00 to 769.70)	<0.001	0.001 (0.0002 to 0.01)	<0.001	0.01 (0.0020 to 0.07)	<0.001
**Had condomless sex during chemsex in the last 3 months (n=31)** [j]										
Baseline	3 (42.86%)			19 (79.17%)			0.20 (31.60 to 1.23)	0.081		
Follow-up	6 (85.71%)	8.00 (0.92 to 69.55)	0.059	22 (91.67%)	2.90 (0.87 to 9.65)	0.082	0.55 (0.04 to 7.49)	0.645	2.76 (0.23 to 32.83)	0.414

[a] Bonferroni adjustment was used.
[b] *P* values were obtained by linear mixed-effects models. The control group was the reference category in the models. Baseline characteristics were not adjusted in the models.
[c] The total score ranges from 7 to 35 with a higher score indicating a higher level of self-efficacy for safe sex.
[d] The total score ranges from 14 to 70 with a higher score indicating a higher level of condom use efficacy.
[e] The total score ranges from 16 to 112 with a higher score indicating a higher level of self-efficacy to resist drug use.
[f] The reference category in the model was "no".
[g] Sequential Bonferroni adjustment was used.
[h] *P* values were obtained by generalized linear mixed-effects models with logit link. The control group was the reference category in the models. Baseline characteristics were not adjusted in the models.
[i] Participants who reported HIV-positive at the baseline assessment were excluded from the analysis.
[j] Only participants who engaged in chemsex in the last 3 months answered the questions about condom use. At the follow-up assessment, only 31 participants had chemsex in the last 3 months.
Abbreviations:
CI: confidence interval; HIV: human immunodeficiency virus; STI: sexually transmitted infection
