# Supplementary material for: Web-Based Harm Reduction Intervention for Chemsex in Men Who Have Sex With Men: Randomized Controlled Trial
Source: JMIR Public Health Surveill. 2023 Jan 5;9:e42902. doi: 10.2196/42902 (PMC9893729; doi:10.2196/42902)
Supplement: Multimedia Appendix 6 [file publichealth_v9i1e42902_app6.pdf]

Multimedia appendix 6: Mixed-effects models for comparison of study outcomes with an adjustment of baseline characteristics

**Primary outcomes**		**Group × Time Effect β (95% CI)**	***P* value** [a, b]
The Self-Efficacy for Sexual Safety Scale		2.12 (0.67 to 3.57)	0.004
The Condom Self-Efficacy Scale			
	Total score	4.53 (2.03 to 7.02)	<0.001
	Consistent Use Subscale	1.08 (0.45 to 1.72)	0.001
	Correct Use Subscale	1.65 (0.54 to 2.75)	0.004
	Communication Subscale	1.80 (0.79 to 2.81)	0.001
The Drug Avoidance Self-Efficacy Scale		6.93 (1.69 to 12.17)	0.010
**Secondary outcomes**		**Group × Time Effect odds ratio (95% CI)**	***P* value** [a, c]
Had chemsex in the last 3 months		0.23 (0.10 to 0.53)	0.001
Intended to have chemsex in the last 3 months		0.37 (0.18 to 0.79)	0.010
Underwent HIV testing in the last 3 months [d]		3.09 (1.72 to 5.56)	<0.001
Underwent other STI testing in the last 3 months		1.47 (0.78 to 2.76)	0.234
Had condomless sex during non-chemsex in the last 3 months (n=31)		N/A [e]	N/A [e]
Had condomless sex during chemsex in the last 3 months (n=31)		N/A [e]	N/A [e]

[a] Only relationship status was adjusted in the models because there was a statistically significant difference in relationship status between the intervention group and control group at baseline assessment.
[b] *P* values were obtained by linear mixed-effects models. The control group was the reference category in the models.
[c] *P* values were obtained by generalized linear mixed-effects models with logit link. The control group was the reference category in the models.
[d] Participants who reported HIV-positive at the baseline assessment were excluded from the analysis.
[e] Due to the small number of participants who engaged in chemsex in the last 3 months (n=31), the adjusted analysis was not conducted for the outcomes.
Abbreviations:
CI: confidence interval; HIV: human immunodeficiency virus; STI: sexually transmitted infection
