# Supplementary material for: Web-Based Harm Reduction Intervention for Chemsex in Men Who Have Sex With Men: Randomized Controlled Trial
Source: JMIR Public Health Surveill. 2023 Jan 5;9:e42902. doi: 10.2196/42902 (PMC9893729; doi:10.2196/42902)
Supplement: Multimedia Appendix 7 [file publichealth_v9i1e42902_app7.pdf]

Multimedia appendix 7: Complete case analysis for comparison of study outcomes (n=275)

	Intervention Group (n=136)	Control group (n=139)	Cohen's *d* effect size	*P* value [b]
**Score difference between follow-up and baseline assessments** [a]	**Mean (SD)**	**Mean (SD)**		
The Self-Efficacy for Sexual Safety Scale [c]	3.49 (6.97)	1.40 (5.54)	0.33	0.006
The Condom Self-Efficacy Scale total score [d]	5.91 (12.65)	1.76 (8.69)	0.38	0.002
The Condom Self-Efficacy Scale Consistent Use Subscale [e]	1.32 (3.07)	0.30 (2.38)	0.37	0.002
The Condom Self-Efficacy Scale Correct Use Subscale [f]	2.29 (5.34)	0.81 (4.19)	0.31	0.011
The Condom Self-Efficacy Scale Communication Subscale [g]	2.30 (5.07)	0.64 (3.59)	0.38	0.002
The Drug Avoidance Self-Efficacy Scale [h]	9.18 (25.87)	2.03 (19.29)	0.31	0.010

	aOR (95% CI)	P value [i]
Had chemsex in the last 3 months (Intervention group vs. control group)	0.11 (0.04 to 0.35)	<0.001
Intended to have chemsex in the last 3 months (Intervention group vs. control group)	0.21 (0.07 to 0.58)	0.003
Underwent HIV testing in the last 3 months (Intervention group vs. control group) [j]	2.21 (1.22 to 3.99)	0.009
Underwent other STIs testing in the last 3 months (Intervention group vs. control group)	1.12 (0.61 to 2.05)	0.719

[a] Subtracting the baseline scores from the follow-up scores
[b] *P* values were obtained by independent *t* tests.
[c] The total score ranges from 7 to 35 with a higher score indicating a higher level of self-efficacy for safe sex.
[d] The total score ranges from 14 to 70 with a higher score indicating a higher level of condom use efficacy.
[e] The subscale score ranges from 3 to 15 with a higher score indicating a higher level of condom use efficacy.
[f] The subscale score ranges from 6 to 30 with a higher score indicating a higher level of condom use efficacy.
[g] The subscale score ranges from 5 to 25 with a higher score indicating a higher level of condom use efficacy.
[h] The total score ranges from 16 to 112 with a higher score indicating a higher level of self-efficacy to resist drug use.
[i] P values were obtained by multiple logistic regression analysis. The corresponding baseline values were adjusted in the model. The control group was the reference category in the models.
[j] Participants who reported HIV-positive at the baseline assessment were excluded from the analysis.
Abbreviations:
aOR: adjusted odds ratio; CI: confidence interval; HIV: human immunodeficiency virus; SD: standard deviation; STI: sexually transmitted infections
